# Non-centrosomal epidermal microtubules act in parallel to LET-502/ROCK to promote *C. elegans* elongation

**DOI:** 10.1242/dev.126615

**Published:** 2016-01-05

**Authors:** Sophie Quintin, Shahoe Wang, Julien Pontabry, Ambre Bender, François Robin, Vincent Hyenne, Frédéric Landmann, Christelle Gally, Karen Oegema, Michel Labouesse

**Affiliations:** 1 IGBMC - CNRS UMR 7104 - INSERM U964 - Université de Strasbourg, 1 rue Laurent Fries, BP 10142, Illkirch 67404, Cedex, France; 2 Ludwig Institute for Cancer Research, Department of Cellular and Molecular Medicine, University of California, San Diego, La Jolla, CA 92037, USA; 3 Institut de Biologie Paris Seine, IBPS FR3631, Université Pierre et Marie Curie, 7-9 Quai Saint Bernard, Paris 75005, France

**Keywords:** *Caenorhabditiselegans*, Morphogenesis, Non-centrosomal microtubules

## Abstract

*C. elegans* embryonic elongation is a morphogenetic event driven by actomyosin contractility and muscle-induced tension transmitted through hemidesmosomes. A role for the microtubule cytoskeleton has also been proposed, but its contribution remains poorly characterized. Here, we investigate the organization of the non-centrosomal microtubule arrays present in the epidermis and assess their function in elongation. We show that the microtubule regulators γ-tubulin and NOCA-1 are recruited to hemidesmosomes and adherens junctions early in elongation. Several parallel approaches suggest that microtubule nucleation occurs from these sites. Disrupting the epidermal microtubule array by overexpressing the microtubule-severing protein Spastin or by inhibiting the *C. elegans* ninein homolog NOCA-1 in the epidermis mildly affected elongation. However, microtubules were essential for elongation when hemidesmosomes or the activity of the Rho kinase LET-502/ROCK were partially compromised. Imaging of junctional components and genetic analyses suggest that epidermal microtubules function together with Rho kinase to promote the transport of E-cadherin to adherens junctions and myotactin to hemidesmosomes. Our results indicate that the role of LET-502 in junctional remodeling is likely to be independent of its established function as a myosin II activator, but requires a microtubule-dependent pathway involving the syntaxin SYX-5. Hence, we propose that non-centrosomal microtubules organized by epidermal junctions contribute to elongation by transporting junction remodeling factors, rather than having a mechanical role.

## Abstract (precis)

**Summary:** During *C. elegans* embryonic elongation, microtubules nucleate at adjerens junctions and hemidesmosomes, and are important for the transport of junctional proteins.

## INTRODUCTION

Morphogenetic processes involve coordinated cell shape changes and movements driven by cytoskeleton-mediated forces, prominently the motor activity of non-muscle myosin II (Lecuit and Lenne, 2007; Quintin et al., 2008; Gorfinkiel and Blanchard, 2011). Recent data suggest that microtubules are also involved. Microtubules are important for the development of tubular organs, in particular for branching morphogenesis and intracellular lumen formation *in vitro* and in *Drosophila* (Gervais and Casanova, 2010; Myers et al., 2011; Gervais et al., 2012; Booth et al., 2014). In addition to centrosomal microtubule arrays that contribute to spindle assembly and cell division, microtubules also form non-centrosomal arrays in differentiated cells that serve structural, mechanical and transport-based functions (Desai and Mitchison, 1997). In epithelial tissues, centrosomes are often inactivated and microtubules become organized from non-centrosomal sites (Bartolini and Gundersen, 2006). In the *Drosophila* trachea and in multiple epithelial tissues in *C. elegans*, the nucleating factor γ-tubulin relocalizes from centrosomes to the cell surface during differentiation (Zhou et al., 2009; Brodu et al., 2010; Fridolfsson and Starr, 2010; Feldman and Priess, 2012; Wang et al., 2015). In vertebrates, the coiled-coil protein ninein, implicated in microtubule anchoring (Dammermann and Merdes, 2002; Delgehyr et al., 2005), also relocalizes to non-centrosomal sites during the assembly of microtubule arrays in epithelial cells. Ninein localizes to the apical cell surface in mouse cochlear epithelial cells (Mogensen et al., 2000; Moss et al., 2007) and to desmosomal junctions required for the assembly of peripheral microtubule arrays in the mouse epidermis (Lechler and Fuchs, 2007). A *C. elegans* protein with homology to ninein, NOCA-1, is required for the assembly of non-centrosomal microtubule arrays in multiple tissues, including the embryonic epidermis (Green et al., 2011; Wang et al., 2015).

**Fig. 1.**
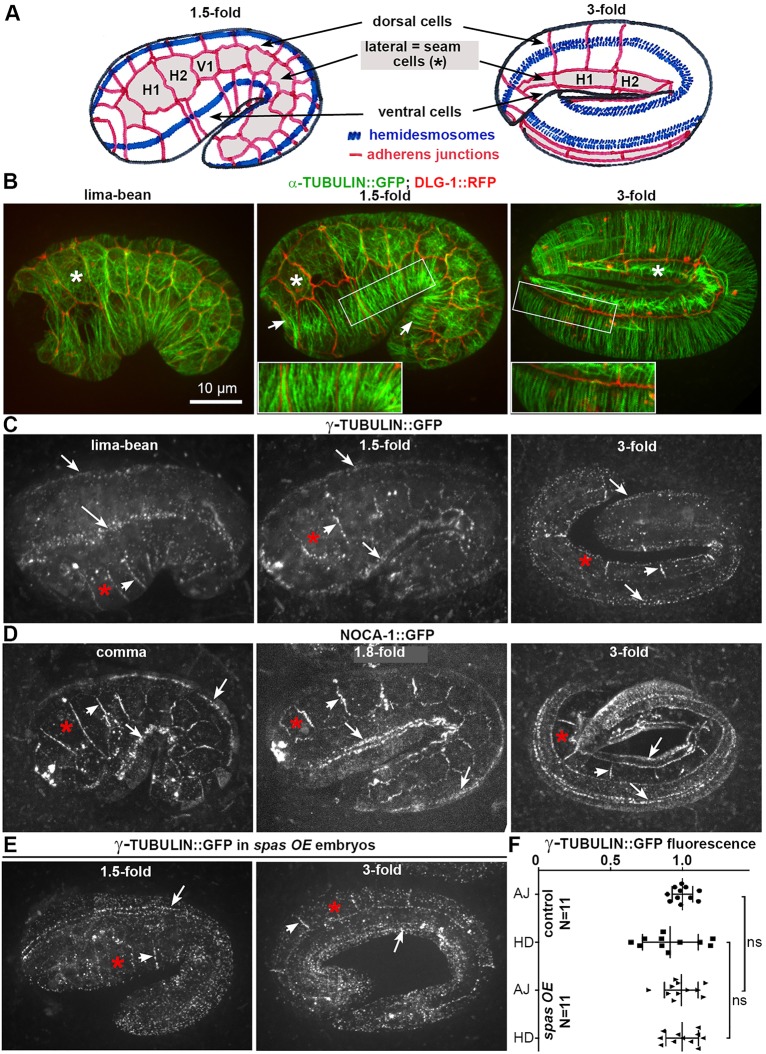
**Epidermal microtubule organization in wild-type embryos.** (A) Schematic drawing of *C. elegans* embryonic epidermis, organized in three rows of cells: dorsal, ventral and seam (gray). Head (H1, H2) and body (V1) seam cells are illustrated. The hemidesmosomes run as four stripes (two on each side) along dorsal and ventral cells. Note the cell shape changes and the punctate-to-stripe hemidesmosome maturation along elongation. In this and subsequent figures, embryos are shown dorsal side up with anterior to the left; asterisk indicates seam cells. (B-D) Spinning-disc confocal lateral projections of wild-type embryos expressing reporters of α-tubulin (TBA-2::GFP) and adherens junctions (DLG-1::RFP) (B), γ-tubulin (TBG-1::GFP) (C) and NOCA-1::GFP (D). Microtubules are enriched along hemidesmosomes (between arrows, middle inset) and come into close vicinity to adherens junctions (right inset). (C) The non-centrosomal γ-tubulin signal and NOCA-1::GFP signal are present along hemidesmosomes (arrows) and seam-seam adherens junctions (arrowheads) throughout elongation; in addition, NOCA-1::GFP becomes distributed all around junctions (3-fold panel). (E,F) The γ-tubulin::GFP signal is not affected in *spas OE* embryos (E), as illustrated by the fluorescence intensity quantification (F). ns, not significant, *P*>0.05; bars indicate mean and s.d. HD, hemidesmosomes; AJ, adherens junctions.

The *C. elegans* embryo offers a powerful model with which to study morphogenesis. Within 3 h, the nematode embryo elongates 4-fold along its anterior-posterior axis with a concurrent reduction in diameter, in the absence of cell division (Chisholm and Hardin, 2005). Elongation requires cell shape changes in the three rows of epidermal cells enclosing the embryo (Fig. 1A) (Sulston et al., 1983). Lateral (or seam) cells are considered to be the major drivers of elongation, since myosin II activity, controlled by the Rho kinase LET-502/ROCK, is predominantly required in these cells (Gally et al., 2009). Dorsoventral cells are juxtaposed to the underlying body wall muscles through transepidermal attachment structures resembling hemidesmosomes (Fig. 1A) (Zhang and Labouesse, 2010). Upon muscle activity, which is essential for elongation beyond the 2-fold stage, dorsoventral cells receive a mechanical signal that promotes hemidesmosome strengthening through intermediate filament phosphorylation (Zhang et al., 2011).

Landmark experiments showed that embryos treated with microtubule inhibitors elongate to a variable extent and often develop bulges, suggesting that microtubules are important for elongation (Priess and Hirsh, 1986). A caveat of drug administration is that elongation defects could result from disrupting microtubules in multiple tissues. Epidermal microtubule arrays could contribute to embryonic elongation via multiple means. We recently developed a continuum mechanics model for elongation that integrates the following parameters: (1) the actomyosin driving force; (2) the hydrostatic pressure exerted by internal cells; and (3) the presence of microtubules in dorsoventral cells, considering their resistance to buckling (Ciarletta et al., 2009). The model predicts that the embryo can elongate provided that microtubules in dorsoventral cells act as a passive mechanical force. Given their role as polarized tracks for cargo transport, an alternative possibility is that microtubules have a transport-based function that promotes elongation.

Here, we investigate how microtubules contribute to *C. elegans* morphogenesis. We sought to determine whether they play a passive mechanical role as predicted by our model, or contribute to morphogenesis via a transport-based function. We show that specifically removing microtubules from the epidermis of *C. elegans* embryos after the 1.5-fold stage only moderately affects elongation, but epidermal microtubules become essential when LET-502 activity is reduced. Additional genetic analysis and imaging of junctional components suggest that, rather than playing a mechanical role, microtubules support elongation by functioning with the syntaxin SYX-5 in parallel to LET-502 to promote the trafficking of E-cadherin to adherens junctions and of the transmembrane receptor myotactin to hemidesmosomes.

## RESULTS

### Non-centrosomal microtubules are nucleated from hemidesmosomes and adherens junctions

To investigate microtubule organization in the epidermis during elongation, we imaged embryos co-expressing the adherens junction marker DLG-1::RFP along with α-tubulin::GFP. Consistent with prior work (Priess and Hirsh, 1986), this analysis revealed that dorsoventral cells contained circumferentially oriented microtubule bundles, whereas the seam cells contained more disorganized microtubule networks (Fig. 1B). At mid-elongation, the region with the most intense tubulin staining coincided with the stripes of hemidesmosomes, which run along dorsal and ventral cells to attach them to the underlying body wall muscles (Fig. 1B, middle inset). To understand how this microtubule array is organized, we examined the localization of GFP fusions with two proteins that have an essential role in the assembly of non-centrosomal microtubule arrays: the microtubule nucleator γ-tubulin and NOCA-1, a *C. elegans* protein with homology to vertebrate ninein (Wang et al., 2015). Both proteins concentrated at hemidesmosomes and at the adherens junctions between seam cells, beginning at the lima-bean stage (Fig. 1C,D). The same localization was observed for a reporter of GIP-2, an essential component of the γ-tubulin small complex (Hannak et al., 2002). The localizations of γ-tubulin (Fig. 1E,F), NOCA-1 and GIP-2 were all microtubule independent (Fig. S1A-D).

**Fig. 2.**
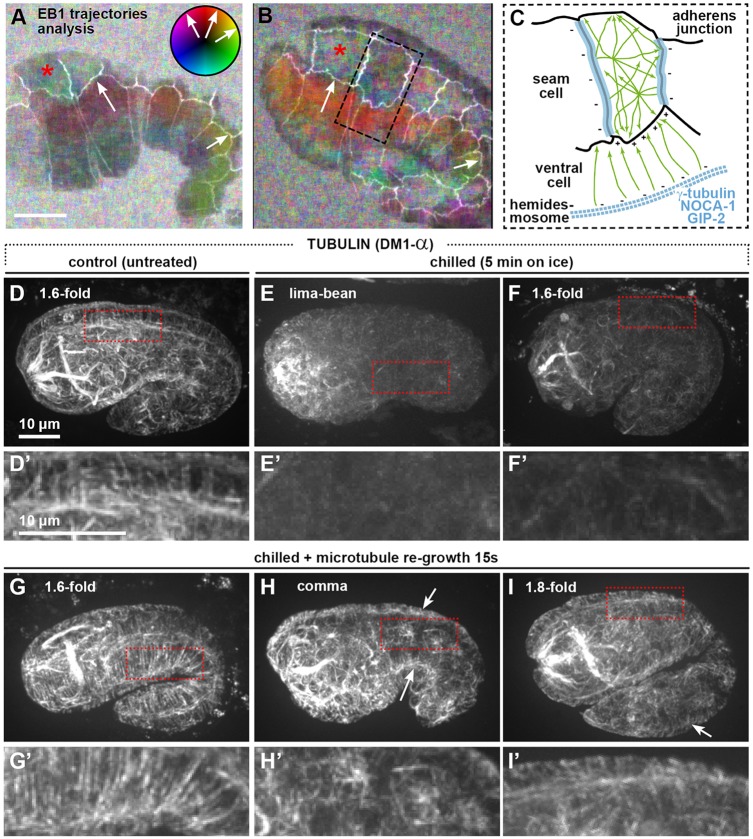
**Microtubules polymerize from hemidesmosomes to seam-dorsoventral cell junctions.** (A,B) ImageJ optical flow analysis deduced from two movies of wild-type embryos co-expressing EBP-2::GFP and DLG-1::RFP reporters (white). The color-coded wheel indicates the orientations of EBP-2 (EB1) trajectories (the predominant color corresponds to that of most EBP-2 comets; black indicates no movement). Microtubules show no preferred orientation in seam cells (multicolor), but grow preferentially towards adherens junctions in ventral cells (arrows). Scale bar: 10 µm. (C) Schematic drawing of microtubule trajectories (green) and minus-end locations (blue) in two adjacent cells (boxed in B). (D-I′) Spinning-disc confocal top projections of wild-type embryos stained by the tubulin antibody (DM1-α) untreated (D), chilled (E,F) or chilled and warmed up for 15 s (G-I). Microtubule regrowth after cold-induced depolymerization seems to be initiated from hemidesmosomes in dorsoventral cells (arrow) or from aster-like foci in seam cells (H,H′). Insets (D′-I′), 2.6× magnification. Scale bar: 10 µm.

Next, we imaged embryos expressing a GFP fusion with the EB1 protein EBP-2, which marks growing microtubule plus-ends (Akhmanova and Steinmetz, 2008). Microtubule growth rates (Fig. S1F) were comparable to those in *C. elegans* zygotes (Srayko et al., 2005). Tracking EBP-2 trajectories revealed that microtubules in dorsoventral cells preferentially grow from the hemidesmosome region towards the seam-dorsoventral junctions, consistent with nucleation from hemidesmosomes, whereas no preferred orientation was observed in the seam cells (Movies 1, 2, Fig. 2A,B). Time-lapse analyses of polymerizing microtubules by total internal reflection fluorescence (TIRF) microscopy also suggested that microtubules grow from the hemidesmosome region, and either from aster-like foci within seam cells or occasionally from seam-seam cell junctions (Movies 3, 4). Consistently, confocal imaging of the embryo outer layer revealed a strong enrichment of epidermal microtubules in the vicinity of hemidesmosomes when microtubules were allowed to briefly regrow (15 s) following cold-induced depolymerization (Fig. 2G-I′). Altogether, our data suggest that in dorsoventral cells in the embryonic epidermis, γ-tubulin, NOCA-1 and GIP-2 are recruited to hemidesmosomes early in elongation, where they direct the nucleation of non-centrosomal microtubules that grow out towards the seam-dorsoventral junctions (Fig. 2C).

### Perturbation of epidermal microtubules leads to mild elongation defects

**Fig. 3.**
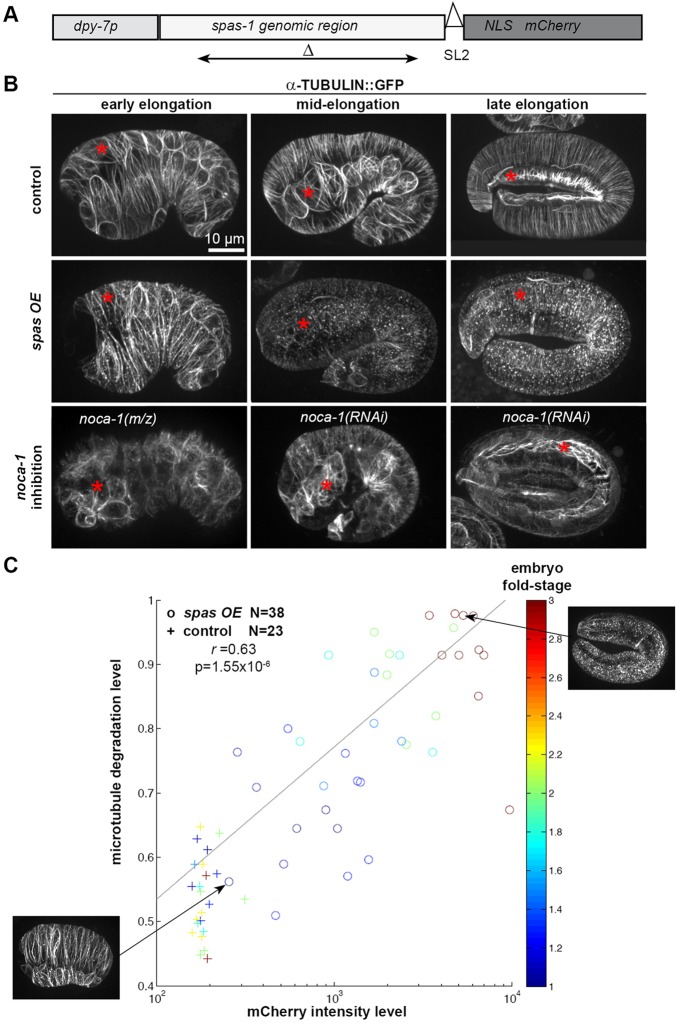
**Spastin expression and *noca-1* inhibition strongly reduce epidermal microtubules.** (A) Schematic drawing of the construct used to drive Spastin (SPAS-1) expression under the epidermal *dpy-7* promoter. An operon linker (spliced leader type 2, SL2) was used. Double-sided arrow indicates the deletion in *spas*Δ (used in Fig. S3); NLS, nuclear localization signal. (B) Spinning-disc confocal lateral projections of embryos expressing the α-tubulin reporter with the indicated perturbations (left). Epidermal Spastin expression leads to a high level of microtubule degradation, starting at mid-elongation. *noca-1* inhibition reduces microtubule levels, especially near adherens junctions and hemidesmosomes. (C) Scatter plot showing microtubule degradation level in *spas OE* embryos (circles) and controls (plus signs) assessed by the appearance of TBA-2::GFP (*y*-axis, maximal microtubule degradation tends to 1 when degraded microtubules appear ‘dotty'; see Materials and Methods) as a function of mCherry intensity (*x*-axis, logarithmic scale). The color-code indicates the fold stage of the embryos analyzed; two examples are shown. Controls have background levels of mCherry and microtubule degradation. Note the correlation between the level of mCherry and microtubule degradation, shown by the Pearson correlation coefficient (*r*) and the low *P*-value: the older the *spas OE* embryos, the more microtubules are degraded.

To examine the contribution of microtubules to morphogenesis, we specifically disrupted them in the epidermis of *C. elegans* embryos using two approaches. First, we forced their disassembly by overexpressing the microtubule-severing protein Spastin (Matsushita-Ishiodori et al., 2007). If Spastin expression was induced by heat shock in early elongation, before the lima-bean stage, embryos failed to elongate. However, once elongation had started, Spastin expression no longer affected morphogenesis (Fig. S2). These results suggest that microtubules might be dispensable for elongation after it has initiated. Consistent with this hypothesis, most transgenic embryos were viable when Spastin was specifically overexpressed in the epidermis using the pan-epidermal *dpy-7* (Gilleard et al., 1997) or *lin-26* (Landmann et al., 2004) promoters, or promoters restricted to dorsoventral (Gilleard et al., 1999) or seam (Cassata et al., 2005) cells (Fig. S2E). As assessed by spinning-disc confocal microscopy, the best level of microtubule degradation was achieved using the *dpy-7* promoter (it becomes increasingly active after the 1.3-fold stage), for which we obtained an integrated line (*spas OE*, for Spastin overexpressed). The construct coupled Spastin expression to that of an mCherry reporter via an operon linker (Fig. 3A). Analyses of α-tubulin::GFP revealed microtubule degradation that started in many 1.5-fold *spas OE* embryos and affected all 1.7-fold *spas OE* embryos and older, but not in embryos expressing an inactive Spastin deleted mutant form (Fig. 3B, Fig. S3C). By contrast, at the earlier lima-bean stage, *spas OE* embryos showed almost no sign of microtubule degradation (Fig. 3B) and a very low level of mCherry, likely reflecting gradual activation of the *dpy-7* promoter. The number of epidermal cells in *spas OE* embryos was unaffected, confirming that microtubule degradation occurred after the birth of epidermal cells (Fig. S3F). Degraded microtubules appeared ‘dotty' rather than linear. Quantification of this feature (see Materials and Methods), confirmed a correlation between Spastin level (reflected by mCherry intensity), microtubule degradation and embryo age (Fig. 3C). In addition, as reported for colcemid-treated embryos (Priess and Hirsh, 1986), several *spas OE* embryos were locally deformed (Fig. S4), suggesting that epidermal microtubules could be required to constrain cell shape, especially in the head.

**Fig. 4.**
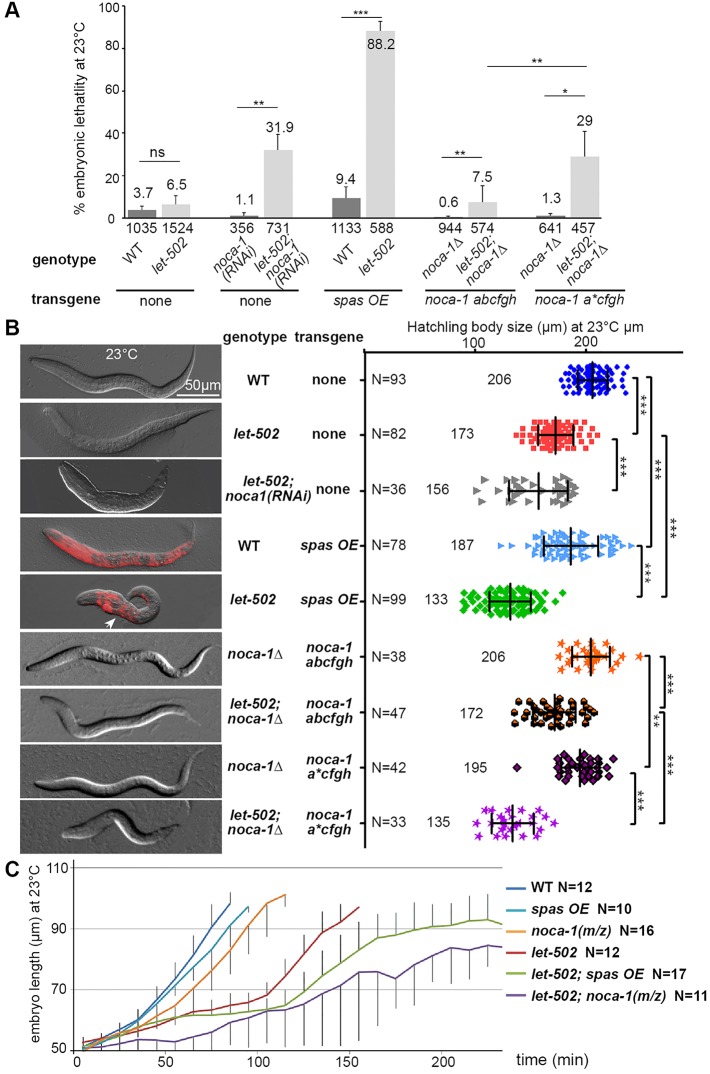
**Microtubule degradation synergizes with a weak *let-502* mutation to impair embryonic elongation.** (A) Bar graph displaying the embryonic lethality in different strains (genotypes as indicated) at the semi-permissive temperature of 23°C for *let-502(sb118ts)*. WT, wild type. (B) DIC images of representative hatchlings and their corresponding size (µm). For *spas OE* animals, the mCherry channel has been superimposed. Note the smaller size of *let-502; spas OE* and *let-502; noca-1*Δ*;* noca-1 a*cfgh animals, and the bulge (arrowhead). Bars indicate mean and s.d. **P<0.001, ***P<0.0001. (C) Elongation curves of these embryos at 23°C, between lima-bean and 2-fold stages.

As a second means of perturbing the microtubule cytoskeleton, we inhibited the function of NOCA-1, which is required for the assembly of non-centrosomal microtubules in the epidermis (Green et al., 2011; Wang et al., 2015). This was achieved in three ways: (1) we analyzed embryos from mothers fed with double-stranded (ds) RNA targeting *noca-1* [termed *noca-1(RNAi)*], and RNAi efficiency was validated as it strongly reduced the NOCA-1::GFP signal (Fig. S1A); (2) we generated embryos homozygous for a deletion removing all *noca-1* isoforms (*noca-1*Δ) and that consequently lack zygotic NOCA-1, and also removed maternal contribution by RNAi [*noca-1 (m/z)*]; and (3) we specifically removed *noca-1* function from the epidermis by rescuing the *noca-1*Δ deletion with either a control transgene expressing six *noca-1* isoforms (*noca-1 abcfgh*) or a transgene lacking the epidermal-specific b isoform of NOCA-1 (noca-1 a*cfgh; Wang et al., 2015). This last approach was useful to rule out contributions to elongation from *noca-1* function in non-epidermal tissues. Consistent with prior work (Green et al., 2011; Wang et al., 2015), analysis of the α-tubulin reporter after *noca-1* inhibition showed a pronounced effect on microtubules (Fig. 3B), yet this triggered almost no embryonic lethality (Fig. 4A).

To investigate the effect of disrupting epidermal microtubules on elongation, we measured hatchling sizes and monitored embryonic development. Both Spastin overexpression and *noca-1* inhibition led to a small but statistically significant effect on worm length at hatching; *spas OE* larvae were ∼10% shorter than their wild-type counterparts and worms lacking the epidermal-specific b isoform (*noca-1*Δ; noca-1 a*cfgh) were ∼5% shorter than controls (*noca-1*Δ*; noca-1 abcfgh*; Fig. 4B). In time-lapse analyses, most *spas OE* and *noca-1(m/z)* embryos developed until hatching but always more slowly than controls (Fig. 4C, Fig. S5). In rare cases (5/55), *spas OE* embryos showed a ventral closure defect, suggesting a requirement for microtubules, reminiscent of *Drosophila* dorsal closure (Jankovics and Brunner, 2006). Such embryos never hatched, presumably accounting for the low level embryonic lethality in the *spas OE* line (9.4%; Fig. 4A). Since Spastin overexpression does not eliminate microtubules before the 1.5/1.7-fold stage, and *noca-1* inhibition severely disrupts epidermal microtubules without completely eliminating them, we cannot rule out their contribution to elongation between the lima-bean and 1.5/1.7-fold stages. However, our data indicate that disruption of the microtubule cytoskeleton after the 1.7-fold stage has only a limited impact on elongation.

### LET-502/ROCK partial loss of function synergizes with microtubule inhibition to cause elongation defects

Our results indicate that epidermal microtubules may facilitate elongation but are not essential after mid-elongation. According to our continuum mechanics model, elongation in the absence of microtubules would become more dependent on the balance of actomyosin forces between dorsoventral and seam cells (Ciarletta et al., 2009). To test this prediction, we lowered myosin II activity by introducing a temperature-sensitive mutation in its upstream activator LET-502, which is enriched in seam cells (Wissmann et al., 1999; Piekny et al., 2003), into the *spas OE* and *noca-1*Δ; noca-1 a*cfgh backgrounds. Whereas most *let-502(sb118ts)* embryos arrest at the 2-fold stage and die at the restrictive temperature of 25°C (Diogon et al., 2007), 93.5% develop normally at the semi-permissive temperature of 23°C (Fig. 4A). By contrast, we observed synergistic embryonic lethality when Spastin was expressed under different promoters in *let-502(sb118ts)* embryos at 23°C (∼90% for *spas OE*; Fig. 4A, Fig. S2E). Hatchling measurements revealed that *let-502; spas OE* embryos grown at 23°C resemble *let-502(sb118ts)* embryos reared at 25°C; at 25°C they were even shorter in length (Fig. S6), with a very low hatching rate (5/50). Synthetic lethality was also observed for *let-502; noca-1*Δ; noca-1 a*cfgh animals, which exhibited 29% lethality at 23°C and a smaller hatchling size than *let-502; noca-1*Δ*; noca-1 abcfgh* animals (Fig. 4A,B). Time-lapse analyses revealed that both Spastin expression and *noca-1* inhibition triggered a 2-fold arrest with a slower elongation rate in the *let-502* background (Fig. 4C, Fig. S5).

The kinase LET-502 could have multiple targets, as often observed for kinases. To determine if LET-502 inhibition enhances loss of epidermal microtubules because of its role in promoting actomyosin contractility, we asked whether *spas OE* would also synergize with a mutation in the myosin regulatory light chain MLC-4, a classical ROCK target (Piekny et al., 2003). *mlc-4* null mutants divide normally due to maternal contribution but cease elongating when they reach the 2-fold stage (Shelton et al., 1999; Gally et al., 2009). We found that *mlc-4(or253); spas OE* embryos arrested at the 2-fold stage like *mlc-4(or253)* embryos, and hence there was no synergistic effect (Fig. S6). In addition, we examined whether the localized expression of a constitutively active form of MLC-4, MLC-4(DD) (Gally et al., 2009), which should bypass the need for LET-502 (Jordan and Karess, 1997; Watanabe et al., 2007), could rescue the elongation defects of *let-502; spas OE* embryos. Using promoters specific for different epidermal cell subtypes to express MLC-4(DD), we did not observe any rescue of viability or hatchling size (Fig. S7B). This result is also consistent with the observation that MLC-4(DD) is unable to rescue *let-502(sb118ts)* mutants, suggesting that MLC-4 is not the only target of LET-502 (Gally et al., 2009). In cell culture, perturbing microtubules impacts myosin II accumulation at cell-cell contacts (Stehbens et al., 2006; Sumigray et al., 2012), prompting us to examine MLC-4::GFP distribution in *spas OE* embryos (Gally et al., 2009). However, microtubule degradation had no apparent effect on MLC-4::GFP localization, and it was not enriched at junctions (Fig. S7C). We conclude that LET-502 most likely exerts its synergistic effects with microtubules through a target other than myosin II.

### Epidermal microtubules promote E-cadherin clustering and turnover at adherens junctions

**Fig. 5.**
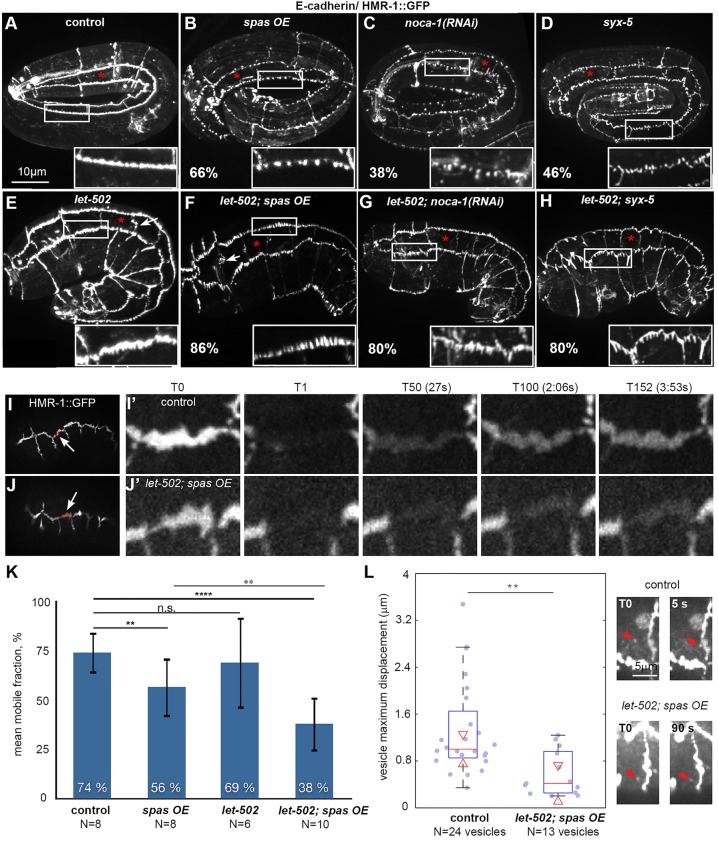
**Microtubules and LET-502 promote adherens junction clustering and E-cadherin mobility.** (A-H) Spinning-disc confocal projections of embryos (genotypes as indicated) expressing the E-cadherin reporter HMR-1::GFP at 25°C. Insets, 2.4× magnification. The penetrance (%) of adherens junctions of fragmented appearance is indicated (*n*≥17). Seam-seam junctions are also highly disorganized (arrows) in *let-502* (E, *n*=11/17) and *let-502; spas OE* (F, *n*=18/20) embryos. (I,J) Images showing the photobleached V1 junction, highlighted in red (arrow). (I′,J′) Insets extracted from FRAP movies, showing that the mutant (J′) does not recover the same intensity as the control (I′). (K) Bar graph showing the mean mobile fraction (with s.d.) of HMR-1::GFP (see Materials and Methods). Note the reduced mobile fraction in *let-502; spas OE* embryos. (L) Box plot displaying the maximum displacement (see Materials and Methods) of intracellular E-cadherin vesicles, as illustrated to the right (arrowheads). Fourteen movies were analyzed in controls and ten in *let-502; spas OE* embryos. Red triangles indicate 95% CI. n.s., non significant (*P*>0.05); **P<0.001, ****P<0.00001.

The observation that a lack of actomyosin tension was unlikely to account for the elongation defects of *let-502; spas OE* embryos suggested that microtubules might not provide a passive force, and prompted us to explore other possible roles. We focused on adherens junctions because they are important for elongation (Costa et al., 1998; Pasti and Labouesse, 2014) and prior work has suggested a functional link between microtubules and adherens junctions (Stehbens et al., 2006; Meng et al., 2008; Bellett et al., 2009; Huang et al., 2011; Sumigray et al., 2012; Revenu et al., 2014). Examination of E-cadherin/HMR-1::GFP (Achilleos et al., 2010) revealed fragmentation of the cadherin-based junctional complex in 38% of *noca-1(RNAi)* and 66% of *spas OE* embryos (Fig. 5B,C). *let-502(sb118ts)* embryos displayed no fragmentation but did exhibit irregular seam-seam junctions (Fig. 5E), which were highly penetrant in *let-502; spas OE* embryos (Fig. 5F). Strikingly, over 80% of *let-502; spas OE* and *let-502; noca-1(RNAi)* embryos showed junctions with marked indentations (Fig. 5F,G). By contrast, we did not observe any major defects in the second adherens junction complex defined by the DLG-1 protein (Fig. S3) (Pasti and Labouesse, 2014).

To further characterize the effects of microtubule inhibition on E-cadherin, we performed fluorescence recovery after photobleaching (FRAP) experiments to determine whether HMR-1::GFP turnover was affected (Fig. S8). We photobleached an entire seam-dorsoventral junction at the 1.7-fold stage, shortly after dorsal cell fusion when embryos do not yet exhibit fragmented junctions, which arise ∼2 h later. Compared with controls (74%), the E-cadherin mobile fraction in *spas OE* embryos was significantly lower (56%) and even lower in *let-502; spas OE* mutants (38%) (Fig. 5I-K, Movie 5). By contrast, E-cadherin turnover in *let-502(sb118ts)* embryos was not significantly different than that in controls. Given the short duration of our FRAP movies (4:30 min), microtubules are likely to promote E-cadherin turnover at adherens junctions by enabling transport from a cellular pool. Consistently, we observed that intracellular E-cadherin vesicles were less mobile in the double mutant (Fig. 5L, Movie 6). Thus, *let-502* and *spas OE* synergize with respect to both elongation and E-cadherin turnover. Since a constitutively active MLC-4(DD) did not rescue elongation in *let-502; spas OE* embryos (Fig. S7), we suggest that LET-502 activity could stimulate junction turnover through a target other than MLC-4.

### Microtubules and ROCK activity promote proper hemidesmosome organization

**Fig. 6.**
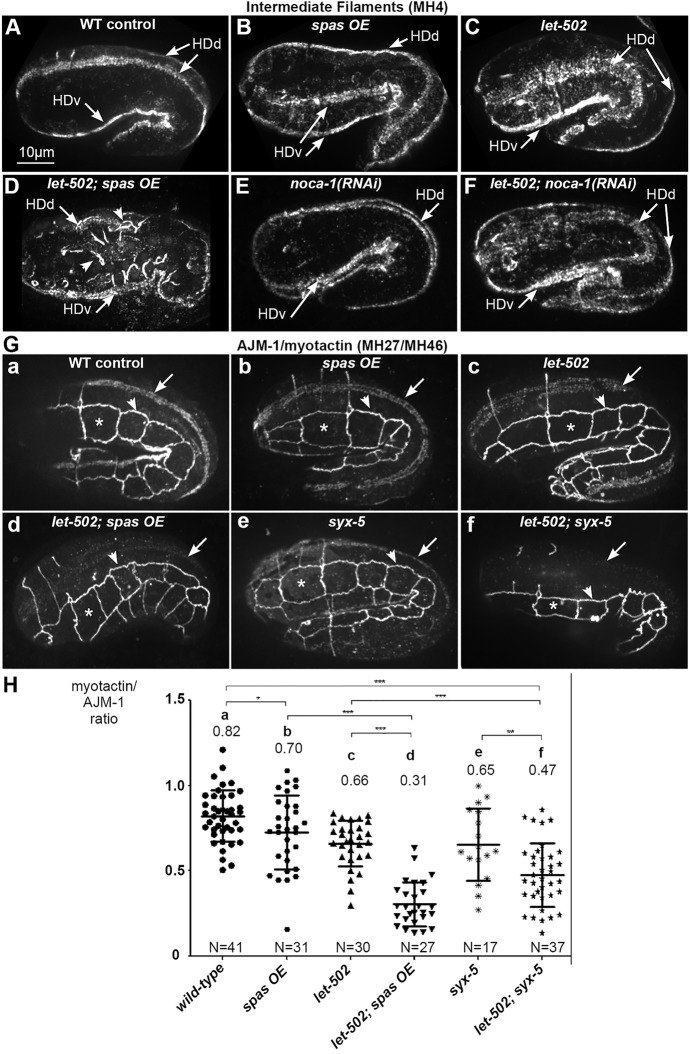
**Microtubules are required for hemidesmosome integrity.** (A-F) Spinning-disc confocal projections of embryos after immunolabeling with the MH4 antibody directed against intermediate filaments present at dorsal and ventral hemidesmosomes (HDd and HDv, arrows). Note the ectopic bundles in *let-502; spas OE* embryos (D, arrowheads, *n*=19/24) and the diffuse pattern in *let-502; noca-1(RNAi)* (F, *n*=12/14) embryos. (G) Spinning-disc confocal projections of embryos co-immunostained with antibodies against the adherens junction component AJM-1 (arrowhead) and the hemidesmosome protein myotactin/LET-805 (arrow). Images were acquired under identical conditions. Note the low amount of myotactin in *let-502; spas OE* (d) and *let-502; syx-5* (f) embryos, relative to the unchanged AJM-1 levels. (H) Scatter dot plot displaying the LET-805:AJM-1 intensity ratio in these embryos (see Materials and Methods). Bars indicate mean and s.d. *P<0.01, **P<0.001, ***P<0.0001.

Hemidesmosomes are required for maintaining epidermal integrity (Bosher et al., 2003). Moreover, they transmit a mechanical signal from the underlying muscles that promotes hemidesmosome remodeling and embryonic morphogenesis (Zhang et al., 2011). Since two regulators that control microtubule assembly, namely the γ-tubulin complex and NOCA-1, localize to hemidesmosomes, we investigated whether microtubule disruption affects these structures. We began by examining intermediate filaments, which are core hemidesmosome components. In early elongation, intermediate filaments were observed at hemidesmosomes, which run along four lines in the epidermis (Fig. 1A, Fig. 6A). The intermediate filament stripes appeared normal in *spas OE* embryos (Fig. 6B), and slightly wider in *let-502* embryos (18/18; Fig. 6C). In *let-502; noca-1(RNAi)* embryos, intermediate filaments showed a diffuse pattern (Fig. 6F), which was also seen in older *noca-1(RNAi)* embryos (8/12; Fig. 6E), suggesting that NOCA-1 or epidermal microtubules could participate in hemidesmosome maintenance. In *let-502; spas OE* embryos, intermediate filaments formed ectopic bundles (Fig. 6D), a phenotype reminiscent of mechanotransduction-defective embryos (Zhang et al., 2011). Younger *let-502; spas OE* embryos did not show defects (10/10 lima-bean), suggesting that the defects arise when the mechanical stress caused by muscle contraction increases. To test whether hemidesmosomes can sustain mechanical stress in microtubule-perturbed embryos, we examined genetic interactions between *spas OE* and a weak allele that affects the Plectin homolog VAB-10A, another core hemidesmosome component (Bosher et al., 2003). Whereas *vab-10(e698)* animals elongate normally, *vab-10(e698); spas OE* embryos stopped elongation, with signs of muscle detachment (Fig. S9C,D). In conclusion, our results suggest that microtubules contribute in a direct or indirect fashion to strengthening hemidesmosomes when muscles become active after the 1.7-fold stage.

Next, we tested whether microtubules affect localization of the transmembrane hemidesmosome component LET-805/myotactin (Hresko et al., 1999). We stained embryos with two monoclonal antibodies directed against the adherens junction protein AJM-1 (internal control) and myotactin (Zahreddine et al., 2010) and measured the ratio between the two signals. Strikingly, *let-502; spas OE* embryos exhibited a myotactin:AJM-1 intensity ratio that was less than half of that in controls (Fig. 6Ha,d), whereas the ratio was only slightly reduced in *spas OE* or *let-502* embryos (Fig. 6Hb,c), again highlighting synergy between *let-502* and microtubule reduction. We conclude that microtubules and LET-502 activity function in parallel pathways to promote myotactin targeting to hemidesmosomes and proper intermediate filament organization.

### A targeted screen for microtubule or transport regulators in elongation

**Fig. 7.**
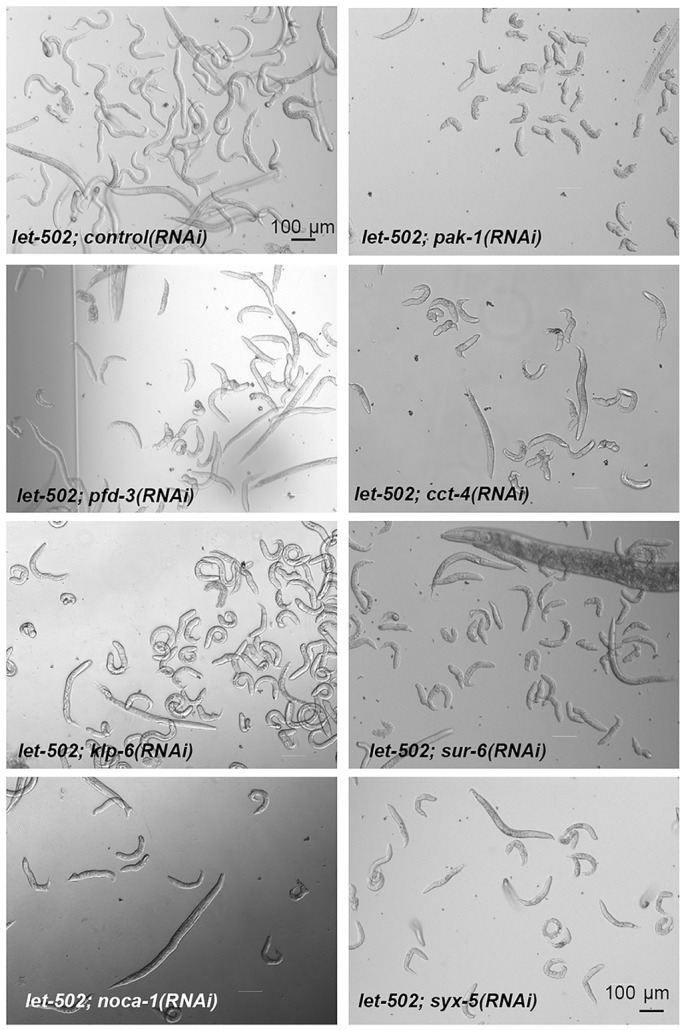
**Knockdown phenotypes from an RNAi screen targeting microtubule or transport regulators in elongation.** DIC images of F1 worms illustrating phenotypes 4 days after RNAi knockdown in wild-type and in *let-502(sb118ts)* backgrounds at 23°C. See Table 1 for a summary of these and further targets.

To identify microtubule-related genes required for elongation, we used the RNAi screen strategy described by Gally et al. (2016). We selected 237 genes related to microtubules and transport (Table S1) to identify enhancers of the *let-502(sb118ts)* mutation at 23°C that would lead to embryonic elongation arrest. With the caveat that we could have missed some potential candidates due to very early embryonic lethality (e.g. *spd-5*, *klp-15*, *zyg-9*), which we could not circumvent using an epidermis-specific RNAi strategy (Qadota et al., 2007), we found 44 hits. Two of them validated the screening strategy, namely *pak-1*, which is known to interact with *let-502* (Gally et al., 2009), and *noca-1* (Fig. 7, Table 1). Other hits encoded proteins in three categories: tubulin folding, centrosome biogenesis, and motors and trafficking. Most genes belonging to the *pfd* and *cct* families significantly enhanced the phenotype of *let-502(sb118ts)* at 23°C (Fig. 7, Table 1). Prefoldins (PFDs) and chaperonin-containing TCP-1 (CCTs) proteins are molecular chaperones important for tubulin folding, depletion of which results in lower tubulin levels and reduced microtubule growth rates (Lundin et al., 2008). Similarly, *let-502* interacted with *evl-20*, which encodes the functional homolog of the human small GTPase ARL2 (Arf-like 2) (40% body morphology defects). In *evl-20(RNAi)* embryos, tubulin concentration is also significantly diminished (Antoshechkin and Han, 2002). These results provide further support for our conclusion that microtubule assembly is crucial when *let-502* activity is reduced. Our screen also identified members of the PP2A complex (SUR-6, RSA-1 and PAA-1), which is essential for centrosome function (Schlaitz et al., 2007), and two kinesin-like proteins (KLP-6 and KLP-10) that might participate in protein transport. The ankyrin UNC-44, which promotes microtubule organization in neurons (Maniar et al., 2012), also appeared as an enhancer of the *let-502(sb118ts)* mutation. Finally, the *syx-5* gene attracted our attention as it triggered one of the highest levels of body morphology defects in combination with *let-502(sb118ts)* (70%; Fig. 7, Table 1)*. syx-5* encodes the ortholog of human syntaxin 5, a member of a protein family regulating membrane fusion events (Hong and Lev, 2014) that could be involved in intracellular transport.

**Table 1.**
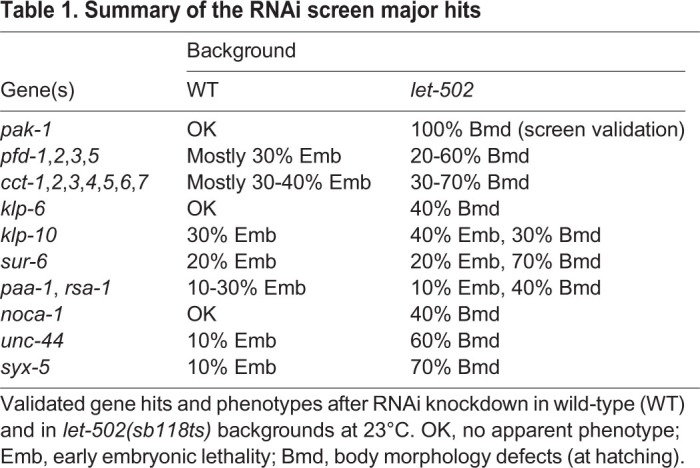
**Summary of the RNAi screen major hits**

### SYX-5 and LET-502 promote myotactin targeting to hemidesmosomes

To further test for genetic interactions between *let-502* and *syx-5*, we used a CRISPR/Cas9-based approach (Dickinson et al., 2013) to generate two mutant *syx-5* alleles, *mc50* and *mc51*, which were both homozygous lethal by the L1/L2 stage (Hyenne et al., 2015). The allele *mc51*, which introduces a frameshift resulting in a premature stop within the T-SNARE domain, was used. In combination with *let-502(sb118ts)*, *syx-5(mc51)* animals were significantly smaller at hatching than single mutants (Fig. S10A). By contrast, loss of *syx-5* function did not reduce *spas OE* size (Fig. S10A), suggesting that *syx-5* functions in a microtubule-dependent pathway. Approximately half of *syx-5(mc51)* embryos beyond 2-fold had E-cadherin clustering defects similar to those in *spas OE* or *noca-1(RNAi)* embryos (Fig. 5D). In *let-502; syx-5* mutants, these defects were highly penetrant, and abnormally long indentations were observed along junctions (Fig. 5H). In addition to adherens junction phenotypes, *let-502; syx-5* embryos showed a significantly reduced myotactin:AJM-1 ratio (Fig. 6Hf), whereas *syx-5* mutants exhibited a wide range of ratios, from normal to highly reduced (Fig. 6He). Lastly, a SYX-5::GFP reporter (Hyenne et al., 2015) showed a punctate pattern throughout the epidermis, possibly the Golgi apparatus, and a faint pattern highly reminiscent of adherens junctions (Fig. S10B). Altogether, these results suggest that the syntaxin SYX-5 functions in a microtubule-dependent pathway that contributes to E-cadherin organization at adherens junctions, myotactin recruitment to hemidesmosomes, and the progression of elongation in parallel to LET-502.

## DISCUSSION

**Fig. 8.**
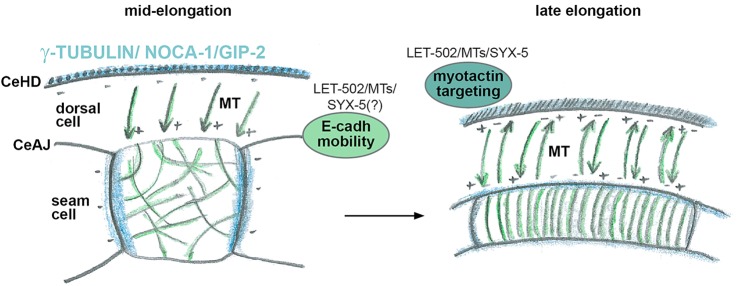
**Model of epidermal** **microtubule and LET-502** **function in *C. elegans* embryo elongation.** Schematic drawing of a seam cell at two different stages. Microtubules (MTs) are depicted in green and minus-end reporter (TBG-1, NOCA-1, GIP-2) localization in blue. A transport mechanism involving microtubules, LET-502 and presumably SYX-5 could ensure E-cadherin transport to adherens junctions (CeAJs) and allow the targeting of myotactin/LET-805 to hemidesmosomes (CeHDs).

Here, we report a characterization of microtubule organization, dynamics and function in the epidermis of elongating *C. elegans* embryos. Our work suggests that microtubules grow out from nucleating sites at hemidesmosomes in the dorsoventral cells towards the adherens junctions with the seam cells. We propose that, beyond the 1.5-fold stage, microtubules are likely to promote elongation by playing a role in the transport of adherens junction and hemidesmosome components (Fig. 8). We further suggest that a microtubule/SYX-5 pathway acts in parallel to a LET-502-dependent pathway to promote junction remodeling, and that LET-502 might exert its function independently of its canonical role in myosin II activation.

### Reorganization of the microtubule cytoskeleton is accompanied by recruitment of the γ-tubulin complex and NOCA-1 to hemidesmosomes

A previous study of the cytoskeleton in elongation showed that microtubules form circumferential bundles in dorsoventral cells and disorganized arrays in the seam cells (Priess and Hirsh, 1986). Here we show that restructuring of microtubule arrays in the dorsoventral cells is accompanied by recruitment of the γ-tubulin complex and NOCA-1 to hemidesmosomes. Recent work has shown that the γ-tubulin complex and the ninein homolog NOCA-1 function together to promote microtubule nucleation and/or stabilization of nascent microtubule minus-ends in non-centrosomal microtubule arrays (Wang et al., 2015). Consistent with this, analysis using the plus-tip marker EBP-2 to track microtubule trajectories revealed that microtubules preferentially grow out from hemidesmosomes towards adherens junctions. Chill and re-warm experiments also suggested that microtubules initiate at hemidesmosomes during recovery following depolymerization. Targeting of ninein homologs to hemidesmosomes/desmosomes to allow them to serve as microtubule-organizing centers might be a conserved phenomenon, since prior work in vertebrate skin has shown that disruption of the desmosomal protein desmoplakin prevents the recruitment of ninein to desmosomes and disrupts the peripheral microtubule array in these cells (Lechler and Fuchs, 2007).

### A role for epidermal microtubules in the transport of junctional components

We found that disrupting the epidermal microtubule array by expressing Spastin under an epidermal promoter reduces the accumulation of myotactin at hemidesmosomes and E-cadherin (HMR-1) mobility, but does not block elongation (embryos reached ∼90% of their normal length). A landmark paper previously showed that embryos exposed to microtubule inhibitors continue to elongate, although not fully (Priess and Hirsh, 1986). Overall, our results are consistent with this study; the somewhat more severe effect on elongation in the prior study could originate from more efficient microtubule degradation at early stages or to the fact that drug treatment disrupts microtubules in non-epidermal tissues that also contribute to elongation.

The essential roles of E-cadherin and myotactin in elongation (Costa et al., 1998; Hresko et al., 1999) suggest that reduction of E-cadherin turnover at adherens junctions and myotactin abundance at hemidesmosomes could account for the elongation arrest in *spas OE* embryos that are partially defective for ROCK activity. In agreement with this notion, it has recently been reported that clathrin and its adaptor complex, which are both essential for elongation and endocytosis, are required for E-cadherin targeting (Gillard et al., 2015). Two studies further illustrate that microtubules could serve a transport function required for junction structure. First, during *Drosophila* tracheal morphogenesis, microtubules are essential to sustain E-cadherin (Shotgun) levels at adherens junctions, and this relies on the apical enrichment of recycling endosomes, which is dynein dependent (Le Droguen et al., 2015). Second, in vertebrate Caco-2 epithelial cells, Nezha (CAMSAP3), a member of the Patronin family of microtubule minus-end-associated proteins, is recruited to adherens junctions and, together with the minus-end-directed motor KIFC3 previously implicated in apical membrane transport, is required for cadherin organization (Noda et al., 2001; Meng et al., 2008). Thus, Nezha-mediated anchorage of microtubule minus-ends at adherens junctions could allow KIFC3 to deliver components affecting junction structure. Our prior work indicated that the NOCA-1/γ-tubulin pathway can either work alone or redundantly with the Patronin family member PTRN-1, depending on the tissue, in promoting the assembly of non-centrosomal microtubules. In the embryonic epidermis, we showed that the NOCA-1/γ-tubulin pathway and not PTRN-1 is crucial (Wang et al., 2015). Putting these together, our results suggest that a mechanism involving transport along microtubules initiated by the γ-tubulin/NOCA-1 pathway, rather than a Patronin family member, could operate at seam-dorsoventral adherens junctions in the *C. elegans* epidermis. Microtubules also promote proper hemidesmosome organization, a relationship that remains poorly understood (Osmani and Labouesse, 2014).

### LET-502/ROCK and microtubules regulate intracellular transport

We found that microtubules become essential for elongation only when LET-502 activity is limiting. Several hypotheses can explain this conditional requirement. One possibility is that microtubule depolymerization reduces actomyosin contractility, which combined with a partial LET-502 activity results in a contractility level lower than the threshold required for normal elongation. We do not favor this possibility because (1) expression of a constitutively active myosin II did not rescue *let-502; spas OE* embryos, and (2) we did not observe synergistic genetic interactions between *spas OE* and an *mlc-4* mutant (encoding myosin II regulatory chain). Moreover, the MLC-4::GFP reporter is not enriched at cell contacts or along hemidesmosomes in *C. elegans* embryos, but instead appears as a ‘dotty' signal in epidermal cells organized along circumferential actin bundles (Gally et al., 2009) (Fig. S7). A second possibility is that *let-502* mutant embryos have abnormal microtubules, explaining the synergy between microtubule depletion and reduced *let-502* activity. Although we did not observe any major tubulin distribution defects (not shown), the microtubule growth rate was reduced by 10% in *let-502* dorsoventral cells and by almost 20% in *let-502* seam cells compared with controls (Fig. S1). This might partially account for the synergy between *let-502* and microtubule-depleted backgrounds. However, it is unlikely to explain it fully, since the synthetic lethality observed in *let-502; spas OE* embryos grown at the semi-permissive temperature was very high. We thus favor a third possibility, namely that microtubules would enable the transport of junctional proteins and that LET-502 may stimulate this process, possibly by phosphorylating one or more proteins controlling intracellular trafficking. Indeed, ROCK-mediated phosphorylation of synapsin I and syntaxin 1 influences synaptic transmission in the rat auditory system (Lontay et al., 2012). Likewise, ROCK can stimulate epidermal growth factor receptor endocytosis by phosphorylating endophilin (Kaneko et al., 2005). We suggest that elongation would stop in the absence of microtubules if such a factor remained unphosphorylated.

Consistent with a role for microtubules in E-cadherin transport, we observed a lower E-cadherin mobile fraction and less mobile intracellular vesicles in *let-502; spas OE* embryos. The finding that a *syx-5* mutation interacts genetically with *let-502* supports a model whereby SYX-5 promotes junction remodeling, possibly by mediating the fusion of E-cadherin vesicles with their target membrane. Importantly, *let-502; syx-5* embryos show normal AJM-1 targeting, so that SYX-5 is unlikely to affect E-cadherin targeting through a global secretion effect. We would argue that SYX-5 is unlikely to be a LET-502 target; otherwise, we would not have observed a genetic interaction between them. The identification of LET-502 targets should help decipher its role in elongation.

Although our data support a microtubule-mediated transport hypothesis in morphogenesis, a mechanical role is not excluded. Indeed, a proportion of *spas OE* embryos showed bulges in their head (Fig. S4). Such deformations, which have been noted previously (Priess and Hirsh, 1986), suggest that microtubules could act as a belt around dorsoventral cells.

### Microtubules promote adherens junction integrity

A striking feature of *let-502; spas OE* mutants is that adherens junction integrity is affected at terminal stages, with a fragmented E-cadherin signal (Fig. 5). In contrast to the loss of the E-cadherin signal observed after microtubule depolymerization in thyroid monolayers or in CHO cells (Yap et al., 1995; Waterman-Storer et al., 2000; Stehbens et al., 2006), we mainly observed short fragments perpendicular to the plane of the membrane. This phenotype might be caused by the observed E-cadherin mobility defects. Alternatively, it could result from a lack of tension applied on adherens junctions. Indeed, a similar fragmentation phenotype has been observed previously when the actin pointed-end-binding protein tropomodulin/UNC-94 was inactive together with a *hmp-1/*α-catenin mutation affecting actin binding (Cox-Paulson et al., 2012), or in the loss of function of the actin-bundling protein EPLIN (Taguchi et al., 2011).

Several studies have established that microtubules can have opposite effects depending on the cellular context. Dynamic microtubules promote the recruitment of myosin IIA (Sumigray et al., 2012) or RhoGEF2 via EB1, necessary for apical constriction (Rogers et al., 2004; Booth et al., 2014). By contrast, in the *Drosophila* embryonic epidermis, dynamic microtubules inhibit RhoGEF2, and thereby ROCK recruitment (Bulgakova et al., 2013). One could speculate that regulatory factors asymmetrically located at microtubule ends might couple the elongation of seam-dorsoventral junctions to the concomitant reduction of seam-seam junctions in *C. elegans* elongation. Uncovering the complex relationship between microtubules, ROCK and myosin II will await further studies.

## MATERIALS AND METHODS

### Strains and genetic methods

*C. elegans* strains (Table S2) were maintained as described (Brenner, 1974). *noca-1*Δ embryos were obtained from heterozygous mothers, in which the *noca-1+* allele was closely linked to an *mCherry* transgene inserted using MosSCI (Frokjaer-Jensen et al., 2008). To obtain *noca-1(m/z)* embryos, heterozygous *mCherry(noca-1+)*/*noca-1*Δ L4 animals were soaked for 24 h in *noca-1* dsRNA, allowed to recover, and non-fluorescent embryos were imaged.

### Generation of plasmids and transgenic strains

To generate the EBP-2::GFP reporter, the *ebp-2* genomic region was cloned with the *dpy-7* promoter and fused to *GFP* in the MosSCI vector pCFJ151 (Frokjaer-Jensen et al., 2008). After injection, the MosSCI line *mcSi53* was isolated and validated by PCR (LGII). The TBG-1::GFP and NOCA-1::GFP reporters were similarly obtained (*ltSi63* and *ltSi246*). To generate the α-tubulin::GFP marker, a PCR fusion strategy (Hobert, 2002) was used to combine the *GFP*, *tba-2* genomic sequence and *lin-26* promoter (4.8 kb). After injection with pRF4 and pAT4.14 (PAT-4::CFP plasmid; gift from B. Williams; Mackinnon et al., 2002) and X-ray integration, *mcIs35* was obtained. Spastin constructs were generated using the Multisite Gateway Pro Kit (Invitrogen). Entry clones contained: (1) promoter; (2) *spas-1* genomic region (2262 bp) or *spas-1*Δ (487 bp); (3) the intergenic region of the *rla-1* operon (265 bp, SL2 in Fig. 3) with the NLS-mCherry sequence. Following recombination, various combinations of promoter-*spas-1*-IRES-NLS-mCherry were generated. The *spas OE* line *mcIs54* was obtained after spontaneous integration on LGX.

### DIC and spinning-disc confocal microscopy acquisitions

Time-lapse DIC images were captured with a Leica DMRXA2 (40× objective) coupled to a Coolsnap HQ (Roper Scientific) camera and a temperature-controlled stage (Linkam PE-120). Embryos were dissected out, washed in M9 and transferred to a 5% agarose pad; the coverslip was sealed with paraffin oil. Stacks of 25 images were acquired every 5 min.

For hatchling measurements, 5-10 hermaphrodites laid eggs overnight. Mothers and larvae were washed away the next day. Hatchlings were mounted in M9 (+0.5 mM levamisole) every 30 min.

Spinning-disc confocal images were acquired 3-4 h after egg laying, with an inverted Zeiss Observer Z1 microscope (100× objective) coupled with an Evolve camera (Photometrics) and a Yokogawa spinning-disc head, monitored by MetaMorph (Molecular Devices).

### Quantification of microtubule degradation

Microtubule degradation was expressed in terms of the spatial organization of the tubulin reporter (varying from lines to dots), based on the shape descriptor:

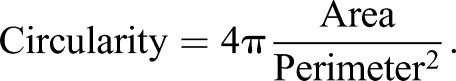


Beforehand, images were subjected to a top-hat transform and a Laplacian operator to highlight microtubules and extract them with an optimal thresholding method (Otsu). For each image, the circularity was averaged over segmented microtubules and the mean mCherry intensity level was measured. These two scalars were reported in a scatter plot of mCherry intensity level against circularity. Using a logarithmic scale, a linear relation appears, quantified with Pearson's correlation coefficient (*r*). Its significance, i.e. the probability that there is no linear correlation, is provided (*P*). Image processing steps were conducted with ImageJ (NIH); statistics with MATLAB (MathWorks).

### Immunostaining of embryos and fluorescence intensity measurements

The microtubule chill and regrowth experiment was performed as described (Hannak et al., 2002). Embryos were fixed and stained as previously described (Bosher et al., 2003). MH4 (anti-intermediate filament), MH46 (anti-LET-805/myotactin) and MH27 (anti-AJM-1) monoclonal antibodies (Developmental Studies Hybridoma Bank) were diluted 1:100, 1:500 and 1:2000, respectively. Intensity measurements were performed using Volocity software (PerkinElmer) by drawing a 90×125 pixel box on embryos and applying a threshold to segment junction and hemidesmosome objects. Average pixel intensity, background subtracted, determined the myotactin:AJM-1 ratio.

### FRAP analyses

1.7-fold stage embryos were imaged at 25°C (Tokai chamber) using a Nikon TiE microscope, equipped with a Yokogawa spinning-disc and a Photometrics Evolve camera piloted by MetaMorph. To avoid *z* drift, the Perfect Focus System was used. Single *z* movies contained pre-bleach acquisitions, a standardized 50 ms photobleach of the entire seam-dorsoventral junction (V1 or H2), and post-bleach acquisitions split in a rapid phase (20 s) and a slower phase (4 min). Owing to embryo movements, intensity measurements were performed manually, background was subtracted, and data were normalized according to Phair et al. (2004). An exponential recovery curve was fitted to the experimental data and the mobile fraction was deduced by extracting the area below the curve (Fig. S8A). Curve fitting and statistical analysis were conducted with MATLAB.

### Vesicle motion analysis

Intracellular E-cadherin vesicles were imaged with the same set-up as in FRAP but using maximum laser power and a frame rate of two images per second. Vesicles were manually selected in lateral cells and automatically tracked using a template-matching method implemented in Fiji. The maximal amplitude of the distances to control points located on neighboring junctions was computed for controls and *let-502; spas OE* mutants. The values were compared using the Wilcoxon signed-rank test.

### Statistical analyses

*P*-values were calculated with either Student's (all other figures) or Wilcoxon (Fig. 5L) tests. Error bars depict s.d. in all figures.

### RNA interference screen

The RNAi feeding screen was performed using the Ahringer library (Kamath et al., 2003) in a 96-well format at 23°C. Missing or erroneous clones were generated manually. Approximately ten L3-L4 stage larvae were placed in each well containing freshly transformed HT115 bacteria producing dsRNAs, resuspended in S-basal. The 96-well plates were kept in a humid chamber for 4 days, analyzed and imaged on a Zeiss AxioPlan microscope (20× objective). For further details see Gally et al. (2016).

### EBP-2 and microtubule dynamics

EBP-2-labeled embryos (ML1654) were imaged at 25°C (Tokai chamber) using an inverted Leica DMI6000 microscope (Yokogawa spinning-disc head) monitored by Andor iQ software. 4D acquisitions included 100 time points of three *z*-planes (0.3 µm step), exposure of 50 ms, no time interval (Movie 2). After each movie, a *z*-stack (DLG-1::RFP) was acquired to locate epidermal cells. Microtubule growth rates were extracted using Volocity (3D-tracking module) and EBP-2 trajectories were analyzed with Fiji.

Microtubule dynamics acquisitions (Movies 3 and 4) were performed using highly inclined and laminated optical sheet (HILO) mode on an Elyra inverted microscope (Zeiss), as previously described (Robin et al., 2014).
